# Improved Performance of Ternary Solar Cells by Using BODIPY Triads

**DOI:** 10.3390/ma13122723

**Published:** 2020-06-15

**Authors:** Sompit Wanwong, Weradesh Sangkhun, Pisist Kumnorkaew, Jatuphorn Wootthikanokkhan

**Affiliations:** 1 Materials Technology Program, School of Energy, Environment and Materials, King Mongkut’s University of Technology Thonburi, 126 Pracha Uthit Road, Bang Mod, Thung Khru, Bangkok 10140, Thailand; weradesh.s@mail.kmutt.ac.th (W.S.); Jatuphorn.woo@kmutt.ac.th (J.W.); 2 National Nanotechnology Center, National Science and Technology Development Agency, 111 Thailand Science Park, Pathum Thani 12120, Thailand; pisist@nanotec.or.th

**Keywords:** BODIPY, P3HT, PCBM, ternary solar cells, power conversion efficiency, morphology

## Abstract

Two boron dipyrromethene (BODIPY) triads, namely **BODIPY-1** and **BODIPY-2**, were synthesized and incorporated with poly-3-hexyl thiophene: (6,6)-phenyl-C61-butyric acid methyl ester (PCBM) P3HT:PCBM. The photovoltaic performance of BODIPY:P3HT:PCBM ternary solar cells was increased, as compared to the control binary solar cells (P3HT:PCBM). The optimized power conversion efficiency (PCE) of **BODIPY-1**:P3HT:PCBM was improved from 2.22% to 3.43%. The enhancement of PCE was attributed to cascade charge transfer, an improved external quantum efficiency (EQE) with increased short circuit current (J_sc_), and more homogeneous morphology in the ternary blend.

## 1. Introduction

A power conversion efficiency (PCE) of bulk heterojunction solar cells of over 13% has been achieved through remarkable progress in the development of narrow gap polymers and non-fullerene acceptors [1,2,3,4,5,6,7,8,9,10,11]. Nevertheless, a single polymer cannot absorb the entire electromagnetic range of the sun spectrum, thus, limiting the amount of photon harvesting capabilities of those solar cells. To solve this issue, tandem solar cells, composed of stacking layers of different chromophores that display complementary absorption to each other, have been demonstrated [1,12,13]. Although tandem cells can improve efficiency up to 17% [14,15], their fabrication process is still complicated and comparatively expensive [12,16,17,18].

Another promising solar cell that can retain the advantages from of bulk heterojunction solar cells and tandem solar cells is the ternary solar cells [19,20,21]. A ternary system offers several advantages including: (a) Light harvesting can be improved by the complementary absorption of the third component. (b) Photovoltaic parameters, short circuit current (J_sc_), open circuit voltage (V_oc_), and fill factor (FF), can be optimized by varying the ratio of donor or acceptor moieties. (c) The morphology and phase separation of the active layer, which play a key role in determining the efficiency, can be adjusted and controlled. (d) The fabrication method is easier than those of multi-layer or tandem solar cells [17,21,22,23,24]. To date, ternary solar cells provide a PCE of up to 12% [21]. Typically, an active layer of ternary solar cells contains a donor (D), an acceptor (A), and a third component. The third component can be either a polymer or a small organic compound that serves as a donor or acceptor. Thereby, ternary solar cells are classified as two donors/one acceptor (D1/D2/A) or and one donor/two acceptors (D/A1/A2) [17,22,25,26]. It has been reported that ternary solar cells based on polymer/small organic molecule/fullerene are more effective than those based on polymer/polymer/fullerene. This is because the latter system tends to have difficulty in controlling crystallinity and phase separation, leading to micrometer scale domain formation [27,28,29].

Small organic compounds are beneficial as they have well-defined structures, precise molecular weight and high purity [1,19,30,31]. Several organic molecules or dyes that show long wavelength absorption and near-infrared (NIR) absorption have been investigated for use in ternary solar cells. In particular, blending small molecules with poly-3-hexyl thiophene (P3HT), a standard donor that has J_sc_ and V_oc_ limits with a band gap of ~2.0 eV, have gained attention. Several groups have blended small organic compounds with P3HT either to improve absorption range or to control phase separation. For examples, An et al. blended a 2-D conjugated small molecule, SMPV1, in P3HT:PC_71_BM and found that SMPV1 can extend the light absorption of the ternary system, resulting in increased photon harvesting in the longer wavelength region. They found that the PCE of ternary solar cells was increased from 3.35% to 4.06%. This was due to the improvement of J_sc_ and FF [32]. Hao et al. mixed a small molecule of p-DTS(FBTTH_2_)_2_ with P3HT:PC_71_BM and found that the best PCE of 3.71% was obtained for p-DTS(FBTTH_2_)_2_:P3HT:PC_71_BM (0.15:0.85:1) ternary solar cells. This PCE value was 24% higher than that of the binary system (P3HT:PC_71_BM). The improvement in PCE is attributed to a combination of cascade charge transfer in the ternary system and Förster resonance energy transfer (FRET) or energy transfer process between P3HT and p-DTS(FBTTH_2_)_2_, leading to the enhancement of J_sc_ values [33]. Honda et al. reported that incorporating silicon phthalocyanine derivative (SiPc) into P3HT:PCBM increased the PCE from 2.2% to 2.7%. They found that when SiPc located at the P3HT/PCBM interface can facilitate energy transfer from P3HT to SiPc molecule, yielding a higher J_sc_ [34]. Haung et al. mixed squaraine (SQ) dyes with P3HT:PCBM. The PCE was increased from 3.27% to 4.51% because the absorption of SQ complemented absorption of P3HT and also overlapped with the photoluminescence (PL) of P3HT. Thus, an efficient FRET process can occur from P3HT to SQ, resulting in the enhancement of both J_sc_ and FF [28]. Wang et al. employed a small diketopyrrolopyrrole based molecule, namely, DPP4T-Cz, as the third component in P3HT:PCBM solar cells. They found that a small amount of DPP4T-Cz (3.4 wt.%) can increase the PCE to 30%, which was attributed to the panchromatic light absorption and energy transfer from P3HT to DPP4T-Cz [35]. Baran et al. incorporate small non-fullerene acceptors, IDTBR and IDFBTR, into a P3HT matrix. The ternary blend P3HT:IDTBR:IDFBTR (1:0.7:0.3) demonstrated the highest PCE of 7.7%. The high performance of the ternary solar cell was due to the optimal phase morphology and reduced charge recombination [36].

Recently, boron dipyrromethene or the BODIPY derivative has attracted attention for enhancing the light absorption in organic solar cells and dye-sensitized solar cells [37,38,39,40,41]. A donor–acceptor–donor (D–A–D) triad is an attractive structure because it can increase cross-sectional absorption and π-conjugation electron delocalization [42,43,44]. The absorption of the donor–BODIPY–donor triad can be tuned from 500 to 800 nm, depending on the structural modification of the BODIPY core and the donor units such as ferrocenyl, benzodithiophene, triphenylamine, and carbazole [42,45,46,47]. Some groups have reported the incorporation of BODIPY in ternary solar cells. For example, Min et al. synthesized two benzannulated aza-BODIPY derivatives for blending with P3HT: (6,6)-phenyl-C61-butyric acid methyl ester (PC_61_BM). Both could extend the absorption of the ternary blends to the near-IR region. However, only aza-BODIPY functionalized with phenyl groups could enhance the PCE from 2.64% to 2.81%. This was attributed to the matching energy level that facilitate cascade charge transfer in the ternary blend [48].

According to our previous report, we synthesized BODIPY triads containing triphenylamine (TPA) and carbazole (CBZ) groups. These BODIPY triads showed an absorption maxima at 540 nm with moderate hole carrier mobilities [49]. Therefore, we are interested to incorporate BODIPYs triads as the second donor in Therefore, we are interested in incorporating BODIPYs triads as the second donor in P3HT:PCBM solar cell. P3HT:PCBM was chosen because P3HT is low-cost and air-processable and has good thermal stability, while PCBM, compared to non-fullerene acceptors, has shown a better photo stability under operational conditions [9,50]. In this work, **TPA-BODIPY-TPA** (**BODIPY-1**) and **CBZ-BODIPY-CBZ** (**BODIPY-2**) were mixed with P3HT with different weight ratios. BODIPY:P3HT donors were blended with fullerene derivative (6,6)-phenyl-C61-butyric acid methyl ester (PC_61_BM), an acceptor, to fabricate ternary solar cells. The photophysical properties of the blended active material were studied. The solar cell efficiency was investigated and the influence of blending BODIPY on the photovoltaic parameter, the external quantum efficiency (EQE) and the surface morphology were discussed.

## 2. Materials and Methods

### 2.1. Materials

(((3,5-Dimethyl-1H-pyrrol-2-yl)(3,5-dimethyl-2H-pyrrol-2-ylidene)methyl)methane) (difluoroborane) and 9-ethylcarbazole-3-boronic acid were purchased from Tokyo Chemical Industry Co. LTD. (Tokyo, Japan). *N*-Iodosuccinimide, 4-(diphenylamino)phenylboronic acid and tetrakis (triphenylphosphine) palladium (0) (Pd(PPh_3_)_4_), poly-3-hexyltiophene (P3HT) (regioregular, 99.995%) with an average molecular weight of 54,000–75,000 g mol^−1^, (6,6)-phenyl-C61-butyric acid methyl ester (PC_61_BM), and indium tin oxide (ITO) coated glass substrates (10 × 10 cm^2^, 6 Ω/cm^2^) were purchased from Merck KGaA (Sigma Aldrich) (Darmstadt, Germany). PEDOT: PSS (PH 1000) was purchased from Ossila Ltd.(Sheffield, UK). TiO_2_ sol-gel was prepared by following the procedure reported by Gregory et al. [51]. Anhydrous sodium sulfate and potassium carbonate were purchased from Thermo Fisher Scientific (Thailand) Ltd. (Bangkok, Thailand). HPLC grade toluene, dichloromethane (DCM), chloroform and hexane were purchased from Fisher. Isopropanol was purchased from Carlo Erba Reagent. DI water (pH 6.2, 0.8 µΩ/cm) was purchased from Siam Beta (Bangkok, Thailand). Deuterated chloroform (CDCl_3_) were purchased from Cambridge Isotope Laboratories. Silica gel for column chromatography was purchased from Silicycle. All chemicals were used as received.

### 2.2. Synthesis

#### 2.2.1. Synthesis of 2,6-diiodo-BODIPY

(((3,5-Dimethyl-1H-pyrrol-2-yl)(3,5-dimethyl-2H-pyrrol-2-ylidene)methyl)methane) (difluoroborane), the BODIPY precursor (0.5 g, 1.9 mmol) was dissolved in chloroform (30 mL) and purged for 10 min. Afterwards, N-iodosuccinimide (NIS) (1.1 g, 4.8 mmol) in dry DMF (6 mL) was added to a BODIPY solution. The reaction mixture was stirred at room temperature under N_2_ gas for 48 h. Next, the solvent was removed from the crude mixture using a rotary evaporator. The crude mixture was then extracted with dichloromethane (DCM) and water. The organic layers were dried over Na_2_SO_4_ and concentrated by rotary evaporator. The crude mixture was purified by column chromatography over silica with DCM/hexane (5:95) as an eluent to yield 2,6-diiodo-BODIPY (0.78 g, 80%). ^1^H-NMR (400 MHz, CDCl_3_): δ (ppm), 2.63 (s, 6H, CH_3_), 2.61 (s, 6H, CH_3_), 2.47 (s, 3H, CH_3_) ^13^C-NMR (100 MHz, CDCl_3_): δ (ppm) 155.06, 142.97, 141.14, 132.14, 85.79, 29.69, 19.83, 19.81, 17.87, 16.00.

#### 2.2.2. Synthesis of BODIPY-1 (TPA-BODIPY-TPA)

2,6-Diiodo-BODIPY (0.18 g, 0.35 mmol) and 4-(diphenylamino)phenylboronic acid (0.25 g, 0.88 mmol) were dissolved in dry toluene (20 mL). The solution was degassed with N_2_ gas for 15 min. Afterwards, Pd(PPh_3_)_4_ (0.04 g, 0.04 mmol) and K_2_CO_3_ (2M) were added to the solution mixture. The reaction mixture was refluxed at 110 °C under nitrogen for 48 h. After the completion of the reaction (checking by TLC), the reaction mixture was extracted with DCM and water. The organic layers were dried over Na_2_SO_4_ and concentrated under reduced pressure. The crude mixture was then purified by column chromatography using DCM/hexane (30:70) as the eluents to yield **BODIPY-1** (**TPA-BODIPY-TPA**) as maroon powder. (1.6 g, 62%). ^1^H-NMR (400 MHz, CDCl_3_): δ (ppm), 7.30–7.26 (m, 8H, CH_AR_), 7.16–7.11 (m, 12H, CH_AR_) 7.08–7.02 (m, 8H, CHAR) 2.71 (s, 3H, CH_3_), 2.53 (s, 6H, CH_3_), 2.38 (s, 6H, CH_3_) ^13^C-NMR (100 MHz, CDCl_3_): δ (ppm), 147.68, 146.79, 131.10, 129.32, 124.58, 123.08, 123.04, 17.30, 15.65, 13.40.

#### 2.2.3. BODIPY-2 (CBZ-BODIPY-CBZ)

2,6-Diiodo-BODIPY (0.20 g, 0.39 mmol) and 9-ethylcarbazole-3-boronic acid (0.20 g, 1.1 mmol) were dissolved in dry toluene (20 mL). The solution was purged with N_2_ gas for 15 min. Next, Pd(PPh_3_)_4_ (0.04 g, 0.04 mmol) and K_2_CO_3_ (2M) were added. After that, the reaction mixture was refluxed at 110 °C under nitrogen for 48 h. The crude mixture was then extracted with DCM and water. The organic layers were dried over Na_2_SO_4_ and concentrated using a rotary evaporator. The crude product was purified by column chromatography using DCM/hexane (30:70) as the eluents to yield **BODIPY-2 (CBZ-BODIPY-CBZ)** as a red-wine colored powder (0.15 g, 59%). ^1^H-NMR (400 MHz, CDCl_3_): δ (ppm), 12 (s, 1H, CH_AR_), 8.10 (s, 1H, CH_AR_), 7.95 (d, 1H, CH_AR_), 7.49–7.45 (m, 6H, CH_AR_), 7.34–7.32 (d, 2H, CH_AR_) 7.27–7.23 (m, 3H, CH_AR_), 4.45 (m, 4H, CH_2_) 2.76 (s, 3H, CH_3_), 2.56 (s, 6H, CH_3_), 2.41 (s, 6H, CH_3_), 1.51 (t, 3H, CH_3_). ^13^C-NMR (100 MHz, CDCl_3_): δ (ppm), 152.70, 141.23, 140.26, 139.27, 137.05 134.43, 132.23, 132.21, 128.13, 125.87, 124.23, 123.05, 122.78, 122.21. 120.48, 118.95, 118.97, 108.59, 108.32, 37.68, 17.26, 15.68, 13.92, 13.44.

### 2.3. Solar Cell Fabrication

The patterned ITO substrates (25 mm × 20 mm) were cleaned with commercial detergent, Alconox solution (10 wt.%), isopropanol, and DI water under sonication for 30 min, respectively. Then, the ITO substrates were treated with UV light (UV-LED, 365 nm, Larson Electronics, Kemp, TX, USA) for 30 min before use. The 80 μL of filtered PEDOT: PSS was deposited onto the ITO substrates by using the spin coating technique at 3000 rpm for 40 s. After annealing at 140 °C for 30 min in air, the substrates were quickly transferred to a glove box filled with nitrogen gas (RH ~10%). The 50 μL of P3HT (and/or **BODIPY-1**, **BODIPY-2**): PCBM (1:1 by wt.) (20 mg/mL in chloroform) solution was spin coated onto the PEDOT: PSS layer at 2500 rpm for 30 s. Then, the 60 μL of TiO_2_ solution was coated on active layer at 4000 rpm for 30 s. The coated substrates were annealed at 110 °C for 10 min and then transferred to thermal evaporator to deposit the aluminum cathode (film thickness ~90–110 nm). The active area of the device was 4 mm^2^.

### 2.4. Instrumentations

^1^H-NMR spectra were recorded on a 400 MHz NMR spectrometer (Ascend 400, Bruker BioSpin GmBH, Rheinstetten, Germany) and were reported in ppm using the residual proton resonance of the solvent as the internal standard (CDCl_3_ at 7.26 ppm). ^13^C-NMR spectra were proton decoupled and recorded on a 100 MHz Bruker spectrometer using the carbon signal of the deuterated solvent as the internal standard. Thermogravimetric analysis (TGA) of **BODIPY-1** and **BODIPY-2** were measured using a thermogravimetric analyzer (Diamond TA/Q50, TA instruments, New Castle, DE, USA). The absorption spectra of BODIPY and P3HT solution were recorded on a Thermo Scientific UV-Genesys 10 s spectrophotometer. The solid state absorption spectra of BODIPY and P3HT and blended films were recorded on a UV-Vis-NIR spectrophotometer (SolidSpec-3700, Shimadzu, Kyoto, Japan). Fluorescence spectra were recorded on a PerkinElmer LS 55 fluorescence spectrometer. Electrochemical Impedance spectroscopy (EIS) (PGSTAT302, Metrohm Autolab B. V., Utrecht, the Natherlands) was conducted on an AUTOLAB. The surface morphology of thin films were characterized using an atomic force microscope (AFM) (NaioAFM, Nanosurf, Liestal, Switzerland). Contact angles were measured by a static optical contact angle meter (KINO SL150E). The current density–voltage (J–V) curves were measured using a Keithley 2400 source-measurement unit and a Newport 94011A solar simulator. The external quantum efficiency (EQE) measurement was performed on a Newport Oriel QEPVSI-b under the irradiation of a Xenon lamp equipped with a monochromator controlled via USB connection through TracQ software (version 6.7, Newport Corporation, Irvine, CA, USA).

## 3. Results and Discussion

### 3.1. Synthesis

**Figure 1:**
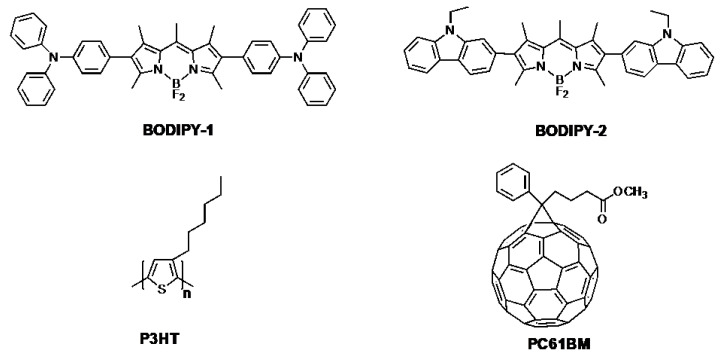
The corresponding chemical structures of boron dipyrromethene-1 (**BODIPY-1)**, **BODIPY-2**, poly-3-hexyl thiophene (P3HT), and (6,6)-phenyl-C61-butyric acid methyl ester (PC_61_BM) for use in ternary solar cells.

Our target BODIPY compounds, **TPA-BODIPY-TPA** (**BODIPY-1**) and **CBZ-BODIPY-CBZ** (**BODIPY-2**) (Figure 1), were synthesized following our previous report [49]. The chemical structures were confirmed using ^1^H-NMR and ^13^C-NMR, respectively (see Supplementary Materials, Figures S1–S6). Thermal gravimetric analysis was carried out to investigate the thermal degradation. The 2.5% decomposition temperatures (T_d_) of **BODIPY-1** and **BODIPY-2** were 200 °C and 225 °C (Figure S7), respectively, indicating that they are stable for use as the active layers of solar cells [52].

### 3.2. Optical Characterizations

**Figure 2:**
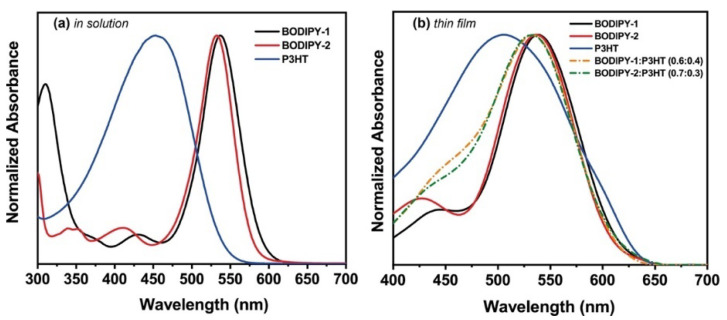
(**a**) Normalized absorption spectra of **BODIPY-1**, **BODIPY-2** and P3HT in chloroform. (**b**) Normalized absorption spectra of **BODIPY-1**, **BODIPY-2**, P3HT, **BODIPY-1**:P3HT (0.6:0.4) and **BODIPY-2**:P3HT (0.7:0.3) films.

The absorption spectra of **BODIPY-1** and **BODIPY-2** exhibited narrow absorption with absorption maxima at 537 and 532 nm, and high extinction coefficients of ~3 × 10^4^ and 8 × 10^4^ M^−1^ cm^−1^, respectively (Figure 2a). P3HT showed a broader spectrum, with absorption maxima at 453 nm, while the solid-state absorption of the P3HT was red-shifted by of ~50 nm with respect to that of the P3HT in solution (Figure 2b). The absorption spectra of BODIPY blended P3HT films are a combination of the individual spectra BODIPY and P3HT, indicating cooperative of light harvesting.

### 3.3. Photovoltaic Performance

**Figure 3:**
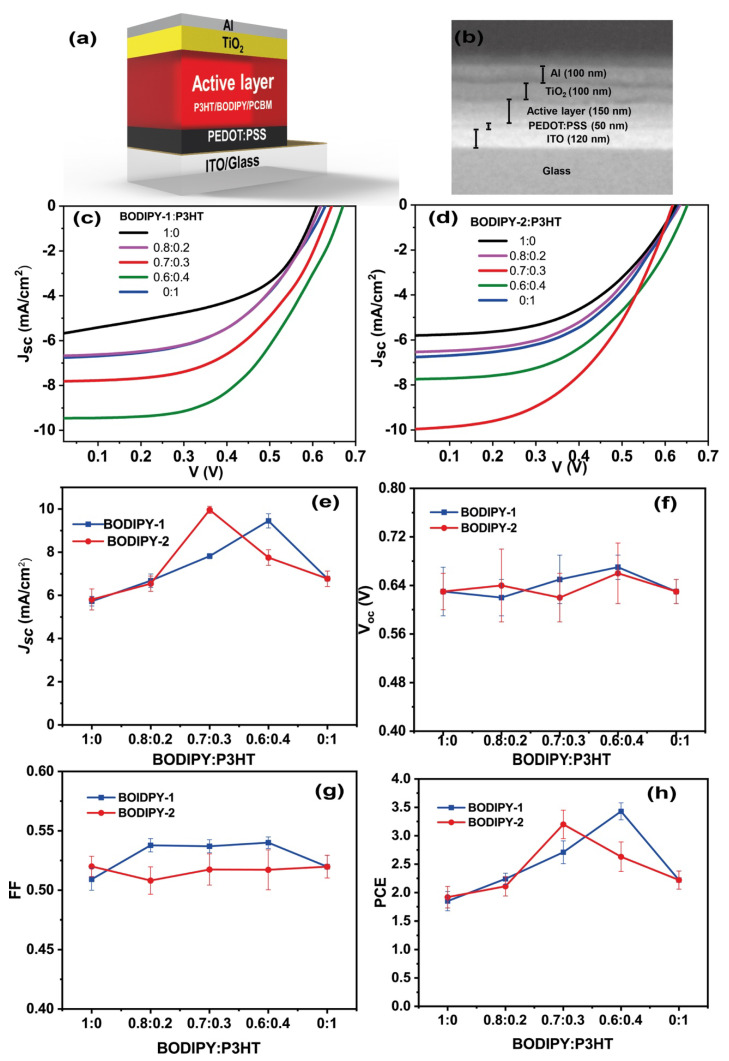
(**a**) Device architecture, (**b**) SEM cross-section of a solar cell, (**c**,**d**) J–V curve of **BODIPY-1**:P3HT:PCBM and **BODIPY-2**:P3HT:PCBM ternary solar cells, and (**e**–**h**) Photovoltaic parameters (J_sc_, V_oc_, FF and PCE) plotted against the weight ratio of BODIPY and P3HT.

Binary and ternary solar cells were fabricated in a regular architecture (ITO/PEDOT:PSS/BODIPY:P3HT:PC_61_BM/TiO_2_/Al) under the same conditions. The current–voltage (J–V) characteristic curves of solar cells are presented in Figure 3c,d and the photovoltaic performance parameters including short circuit current density (J_sc_), open circuit voltage (V_oc_), fill factor (FF) and power conversion efficiency (PCE) are summarized in Table 1. The control device fabricated from P3HT:PCBM provided a PCE of 2.22%, with a J_sc_ of 6.77 mA/cm^2^, a V_oc_ of 0.63 V, and an FF of 0.52. Binary solar devices fabricated from **BODIPY-1**:PCBM and **BODIPY-2**:PCBM have PCEs of 1.85% and 1.92%, respectively. The lower performance of BODIPY:PCBM solar cells is attributed to the narrow absorption properties of BODIPY, resulting in lower J_sc_ values. The photovoltaic performance of ternary solar cells were optimized by varying weight ratio of P3HT and BOIDPY. We found that incorporating a lower quantity of BODIPY in P3HT:PCBM led to a reduction in the performance of devices (see Table S1). Interestingly, increasing BODIPY concentration in P3HT:PCBM significantly enhanced J_sc_ values, leading to the improvement of device efficiency as compared to those of P3HT:PCBM or BODIPY:PCBM binary solar cells (Table 1 and Figure 3e). Ternary solar cells containing **BODIPY-1**:P3HT (0.6:0.4) exhibited the highest PCE (3.43%) due to a large enhancement in J_sc_ (9.45 mA/cm^2^) and an increase in FF (0.54). The efficiency was raised by 55% as compared to the control P3HT device. Incorporating **BODIPY-2** with P3HT also improved the solar cell performance. The device fabricated with **BODIPY-2**:P3HT (0.7:0.3) provided a PCE of 3.20%, with the highest J_sc_ value of 9.96 mA/cm^2^. However, the V_oc_ values of the champion devices are slightly increased as compared to the P3HT device. In general, the V_oc_ of the P3HT:PCBM solar cells is limited by a high voltage loss (ΔE_loss_), in a range of 1.10–1.35 eV [11,53,54,55,56]. This loss is caused by three factors: (i) the radiative recombination from the absorption above the bandgap (~0.30 eV), (ii) an additional radiative recombination from the absorption below the bandgap (>0.6 eV), and (iii) the non-radiative recombination (>0.3 eV) [11,53,54,55,56]. In this work, P3HT:PCBM binary solar cell has a voltage loss of 1.26 eV. Whereas, the voltages loss values of **BODIPY-1**:P3HT (0.6:0.4) and **BODIPY-2**:P3HT (0.7:0.3) ternary solar cells are 1.12 eV and 1.10 eV, respectively. It should be noted that PCE of champion cells are higher than those of reported small BODIPY molecule used in ternary solar cells [48,57]. This suggested that our BODIPY triads and P3HT are a suitable combination to improve the efficiency of ternary solar cells.

### 3.4. External Quantum Efficiency (EQE)

**Figure 4:**
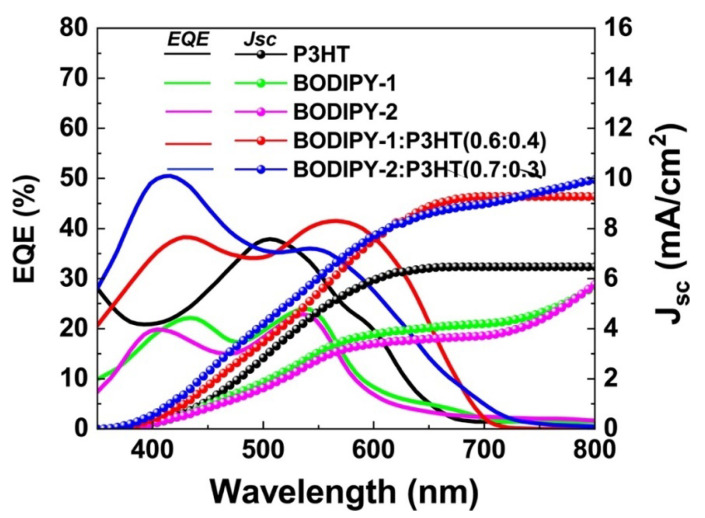
The external quantum efficiency (EQE) spectra of P3HT, **BODIPY-1**, and **BODIPY-2** binary solar cells and **BODIPY-1**:P3HT(0.6:0.4) and **BODIPY-2**:P3HT (0.7:0.3) ternary solar cells.

The EQE spectra of binary solar cell and ternary solar cell are depicted in Figure 4. The EQE of P3HT device was 35% at 500 nm, while the EQE of **BODIPY-1** and **BOIDPY-2** devices were 20% at wavelengths of 400 and 550 nm. For the ternary blend system, the EQE of the ternary solar cells were higher than those of BODIPY and P3HT binary solar cells. The EQE spectra for **BODIPY-1**:P3HT (0.6:0.4) and **BODIPY-2**:P3HT (0.7:0.3) illustrated that EQE in BODIPY and P3HT absorption were increased up to 40% and covered in a broader range from 350 to 700 nm. In addition, the trend in EQE spectra and increase in the photocurrent generation are in good agreement.

### 3.5. Impedance Analysis

**Figure 5:**
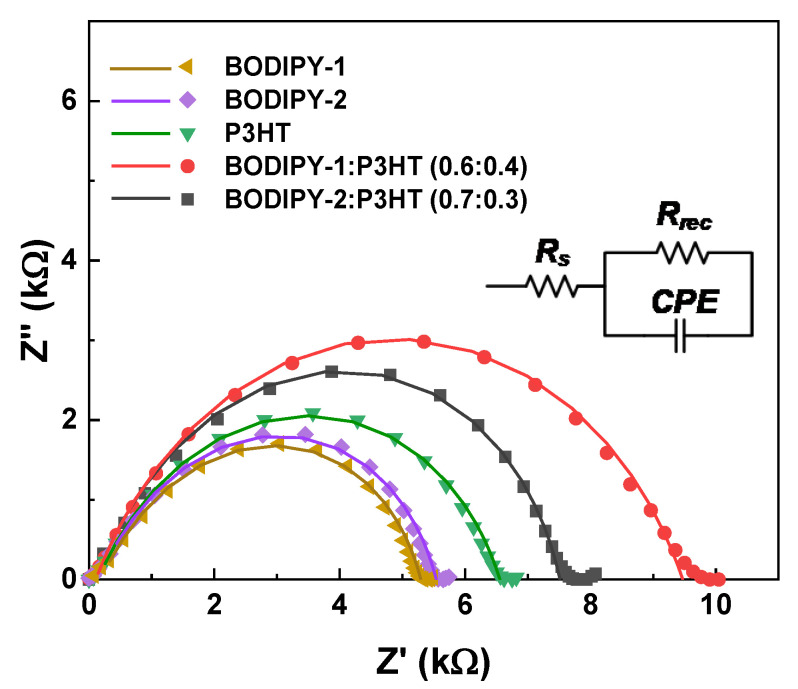
The electrochemical impedance spectroscopy (EIS) spectra of **BODIPY-1**, **BODIPY-2**, and P3HT binary solar cells and **BODIPY-1**:P3HT (0.6:0.4) and **BODIPY-2**:P3HT (0.7:0.3) ternary solar cells under 1 sun simulation and an open circuit condition.

It is well known that the suppression of dark saturation current density (J_0_) can increase the V_oc_ value of organic photovoltaics (OPVs) as a result of the reduction in charge recombination at the interface between the active layer and the anode/cathode layer [58,59]. Electrochemical impedance spectroscopy (EIS) was used to investigate the charge recombination process, which is directly related to the V_oc_ in OPV devices. EIS was performed over a frequency range from 0.01 Hz to 1 MHz at an amplitude of 10 mV. The EIS spectra were fitted by using an equivalent circuit model, as shown in the inset of Figure 5. R_s_, R_rec_, and CPE represented the series resistance, the charge recombination resistance, and the constant phased element representing the chemical capacitance, respectively [60,61]. Figure 5 showed Nyquist plots of binary solar cells and ternary solar cells under 1 sun simulation. The semicircle represented the charge recombination resistance (R_rec_) of the solar cell. It was found that the radius of the semicircle of the Nyquist plot was increased when BODIPY triads were applied in the ternary system. This suggests that charge recombination was significantly suppressed, resulting in the increase in V_oc_ that was observed in champion devices. In addition, we found that the P value of CPE, simulated by an EIS software analyzer (Table S2, increased from 0.70 (P3HT device) to 0.90 (**BODIPY-1**:P3HT (0.6:0.4) device). This P value of the champion device is close to the *P*-value of an ideal capacitor (*P* = 1) without grain boundary and/or defect [59]. Since voltage loss values of **BODIPY-1**:P3HT and **BODIPY-2**:P3HT devices were similar (1.1 eV), a higher PCE of **BODIPY-1**:P3HT could be due to efficient charge transport in the active layer. This suggests that the active layer film formed by the ternary blended system has low charge trapping sites, originating from defects such as pin-holes or the grain boundaries of the film, and corresponds to the atomic force microscope (AFM) results), showing that the quality of the active film was higher and had greater homogeneity when BODIPY triads were applied in the active layer.

### 3.6. Morphology Study

**Figure 6:**
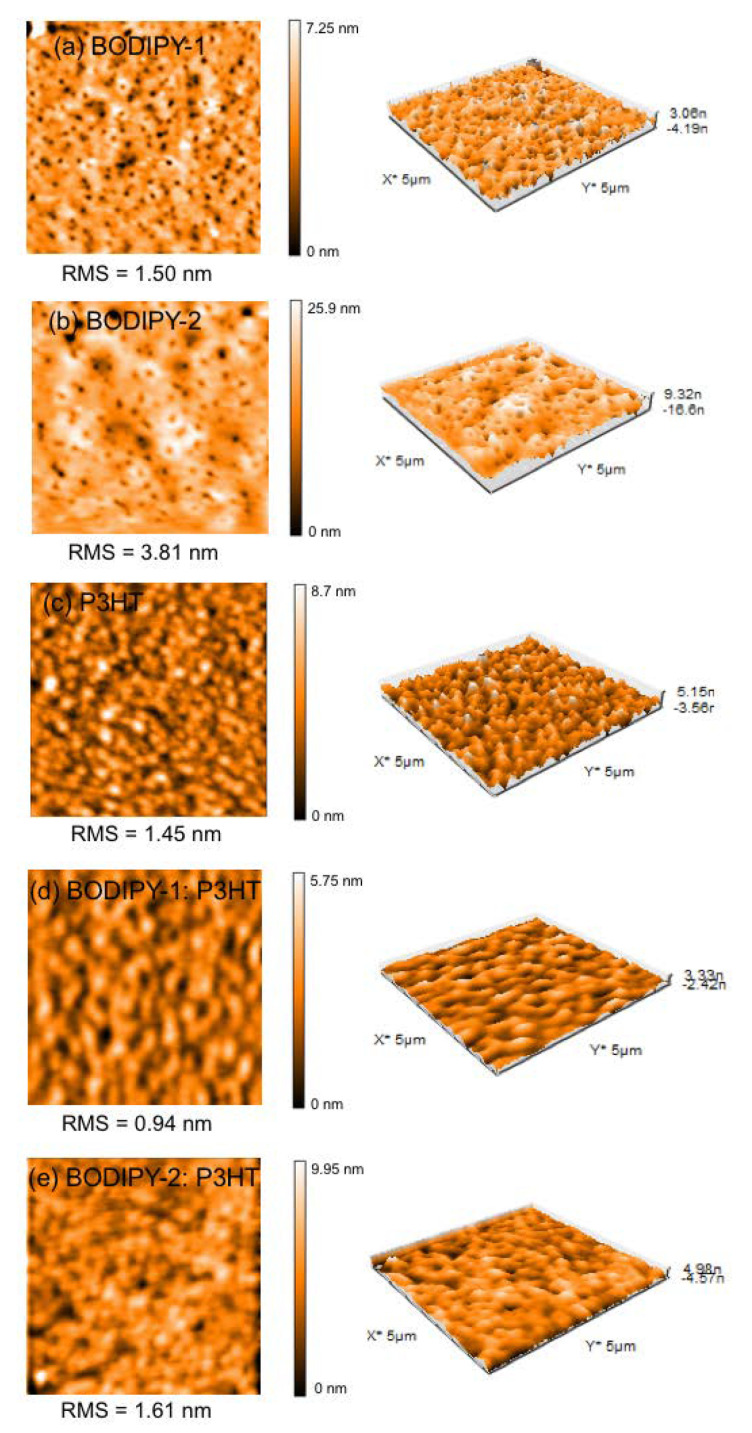
Tapping-mode atomic force microscope (AFM) images (5 μm × 5 μm): (**a**) **BODIPY-1**:PCBM (1:1) film, (**b**) **BODIPY-2**:P3HT (1:1) film, (**c**) P3HT:PCBM (1:1) film, (**d**) **BODIPY-1**:P3HT:PCBM (0.6:0.4:1) film, and (**e**) **BODIPY-2**:P3HT:PCBM (0.7:0.3:1) film.

Morphology of the photoactive layer is known as a critical factor that optimizes photon/electron conversion efficiency of ternary solar cells [62,63,64,65]. To determine surface roughness and phase behavior of the blended active layer, AFM has been investigated in tapping mode (Figure 6a–e). For an accurate comparison, all films were prepared with the same conditions for solar cell device fabrication. It is clearly seen that **BODIPY-1**:PCBM and **BODIPY-2**:PCBM films showed some void (pin-holes) and microphase separation (Figure 6a,b). The surface roughness of BODIPY blended with P3HT:PCBM exhibited lower root mean square of average height (RMS) values compared to those of the binary blends (Figure 6d,e). This indicated that blending BODIPY and P3HT yields a flatter and smoother surface. The RMS values of **BODIPY-1** blended P3HT:PCBM and **BODIPY-2** blended P3HT:PCBM were 0.94 nm and 1.61 nm, respectively. It should be noted that the champion device, **BODIPY-1**:P3HT:PCBM (0.7:0.3:1) developed a more homogeneous film with an improved morphology over the binary counterpart device. This could result in more efficient charge transport at the interfacial of the donor/acceptor area and enhance the PCE [59,66,67,68].

Next, the BODIPY distribution in the ternary blend film was investigated using contact angle measurements of pure water [35,69]. The contact angles of **BODIPY-1**, **BODIPY-2**, P3HT, and PCBM were 93.6°, 83.4°, 95.3°, and 89.9° which led to estimate their surface energy (γ) values of 26.7, 32.4, 24.8, and 29.9 mJ/m^2^, respectively [70,71,72]. The location of BODIPY in the P3HT:PCBM matrix can be estimated in terms of the wetting coefficient (ω) using surface energy and Neumann’s and Young’s equations [69,70,71,73]. According to the published literature [35,61,69,74], if ω > 1, BODIPY will be located in the P3HT domain. If −1 < ω < 1, BODIPY will be located at the interface of P3HT and PCBM. If ω < −1, BODIPY will be located in the PCBM domain. We found that the ω value of **BODIPY-1** was 0.25, which indicates that **BODIPY-1** molecules preferentially locate at the P3HT/PCBM interface. The ω value of **BODIPY-2** was −1.96, which suggests that **BODIPY-2** molecules are likely to be located in the PCBM domain.

### 3.7. Proposed Operating Mechanism

**Figure 7:**
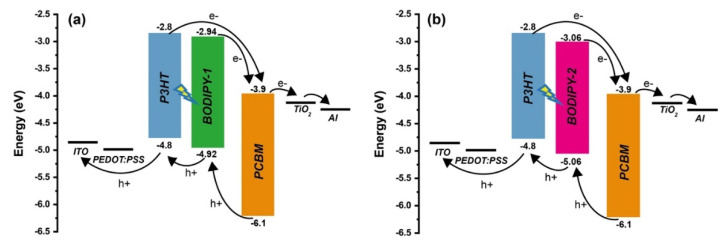
Energy alignment diagram of active material in ternary solar cells. (**a**) **BODIPY-1**:P3HT:PCBM and (**b**) **BODIPY-2**:P3HT:PCBM.

**Figure 8:**
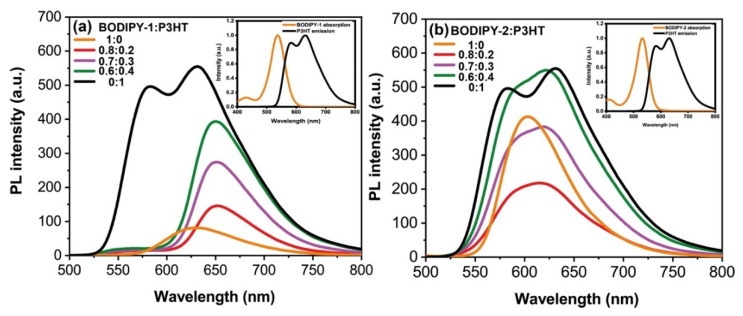
PL spectra of (**a**) P3HT/**BODIPY-1** co-solution in chloroform and (**b**) P3HT/**BODIPY-2** co-solution in chloroform. All samples were excited at 450 nm. Inset: absorption spectrum of BODIPY (orange line) and emission spectrum of P3HT (black line).

There are four fundamental operating models in ternary solar cells: the charge transfer, the energy transfer, the parallel-linkage, and the alloy model [17,18,19,23,26]. Here, we discussed a possible operating mechanism of BODIPY:P3HT:PCBM that enhance J_sc_. Figure 7 presents the energy diagram of donor and acceptors. The HOMO and LUMO energy levels of **BODIPY-1** and **BODIPY-2** were estimated from our previous study [49]. The HOMO and LUMO offset (ΔE) values between P3HT and BODIPY are about ~0.1–0.2 eV, which are energetically feasible in term of driving the cascade charge transfer from P3HT to BODIPY and from BODIPY to PCBM [19,20]. Since P3HT can act as both a charge donor and an energy donor [28,75], we also investigate energy transfer process using a photoluminescence (PL) study. The PL spectra of blending P3HT and BODIPY revealed that P3HT emission can be quenched by a high BODIPY content (Figure 8). This implies that an energy transfer from P3HT to BODIPY was also presented. The energy transfer from P3HT to BODIPY molecules is likely favorable due to the substantial overlapping of the P3HT emission spectrum and the BODIPY absorption, as shown in the inset of Figure 8. Therefore, the boosted J_sc_ is attributed to cascade charge transfer and energy transfer and to the favorable morphology of the ternary blend.

## 4. Conclusions

In summary, we demonstrated a 55% enhancement in PCE in ternary solar cells (**BODIPY-1**:P3HT:PCBM), reaching 3.43%. The improvement in PCE is attributed to efficient charge transfer, and a smooth surface in the ternary blend that facilitates exciton dissociation and charge transportation. This finding suggests that **BODIPY-1** and **BODIPY-2** are suitable small molecules for improving the efficiency of the P3HT:PCBM system.

## Figures and Tables

**Table 1:** Performance parameters of ternary solar cells with BODIPY:P3HT:PCBM.

Devices	Donor Concentration ^a^ (by wt.)	J_sc_ (mA/cm^2^)	V_oc_ (V)	FF	PCE (%) (Best) (Average)
1	P3HT	6.77 ± 0.36	0.63 ± 0.02	51.98 ± 0.96	2.48, 2.22 ± 0.16
2	**BODIPY-1**	5.73 ± 0.22	0.63 ± 0.04	50.91 ± 0.92	2.00, 1.85 ± 0.17
3	**BODIPY-2**	5.81 ± 0.49	0.63 ± 0.03	52.01 ± 0.85	2.29, 1.92 ± 0.19
4	**BODIPY-1**:P3HT (0.8:0.2)	6.68 ± 0.31	0.62 ± 0.01	53.78 ± 0.56	2.33, 2.24 ± 0.10
5	**BODIPY-1**:P3HT (0.7:0.3)	7.82 ± 0.12	0.65 ± 0.04	53.70 ± 0.55	2.90, 2.71 ± 0.20
6	**BODIPY-1**:P3HT (0.6:0.4)	9.45 ± 0.33	0.67 ± 0.02	54.00 ± 0.48	3.71, 3.43 ± 0.15
7	**BODIPY-2**:P3HT (0.8:0.2)	6.54 ± 0.35	0.64 ± 0.06	50.81 ± 1.15	2.23, 2.11 ± 0.17
8	**BODIPY-2**:P3HT (0.7:0.3)	9.96 ± 0.16	0.62 ± 0.04	51.74 ± 1.32	3.38, 3.20 ± 0.25
9	**BODIPY-2**:P3HT (0.6:0.4)	7.75 ± 0.36	0.66 ± 0.05	51.71 ± 1.68	2.99, 2.63 ± 0.26

^a^ All donors were blended with PCBM at a weight ratio of 1:1. The average parameters were obtained from 10 devices.

## Supplementary Materials

The following are available online at https://www.mdpi.com/1996-1944/13/12/2723/s1, Figure S1: ^1^H-NMR of 2,6-diiodo-BODIPY, Figure S2: ^13^C-NMR of 2,6-diiodo-BODIPY, Figure S3: ^1^H-NMR of TPA-BODIPY-TPA (**BODIPY-1**), Figure S4: ^13^C-NMR of TPA-BODIPY-TPA (**BODIPY-1**), Figure S5: ^1^H-NMR of CBZ-BODIPY-CBZ (**BODIPY-2**), Figure S6: ^13^C-NMR of CBZ-BODIPY-CBZ, Figure S7: TGA of **BODIPY-1** and **BODIPY-2**, Table S1: Performance parameters of ternary solar cells with BODIPY:P3HT:PCBM (P3HT concentration was higher than BODIPY), Table S2: Parameters fitted from the Nyquist plot of binary solar cells and ternary solar cells. Table S3: Contact Angle measurement of P3HT, **BODIPY-1**, **BODIPY-2** and PCBM,
