# Supplementary material for: Improved Performance of Ternary Solar Cells by Using BODIPY Triads
Source: Materials (Basel). 2020 Jun 15;13(12):2723. doi: 10.3390/ma13122723 (PMC7344652; doi:10.3390/ma13122723)
Supplement: Supplementary file 1 [file materials-13-02723-s001.pdf]

Supplementary Materials

Improved Performance of Ternary Solar Cells by Using BODIPY Triads

Sompit Wanwong[a]*, Weradesh Sangkhun[a], Pisist Kumnorkaew[b] and Jatuphorn Wootthikanokkhan[a]

[a]*Materials Technology Program, School of Energy, Environment and Materials, King Mongkut's University of Technology Thonburi, 126 Pracha Uthit Road, Bang Mod, Thung Khru, Bangkok 10140, Thailand.*

[b]*National Nanotechnology Center, National Science and Technology Development Agency, 111 Thailand Science Park, Pathum Thani 12120, Thailand.*

NMR spectra

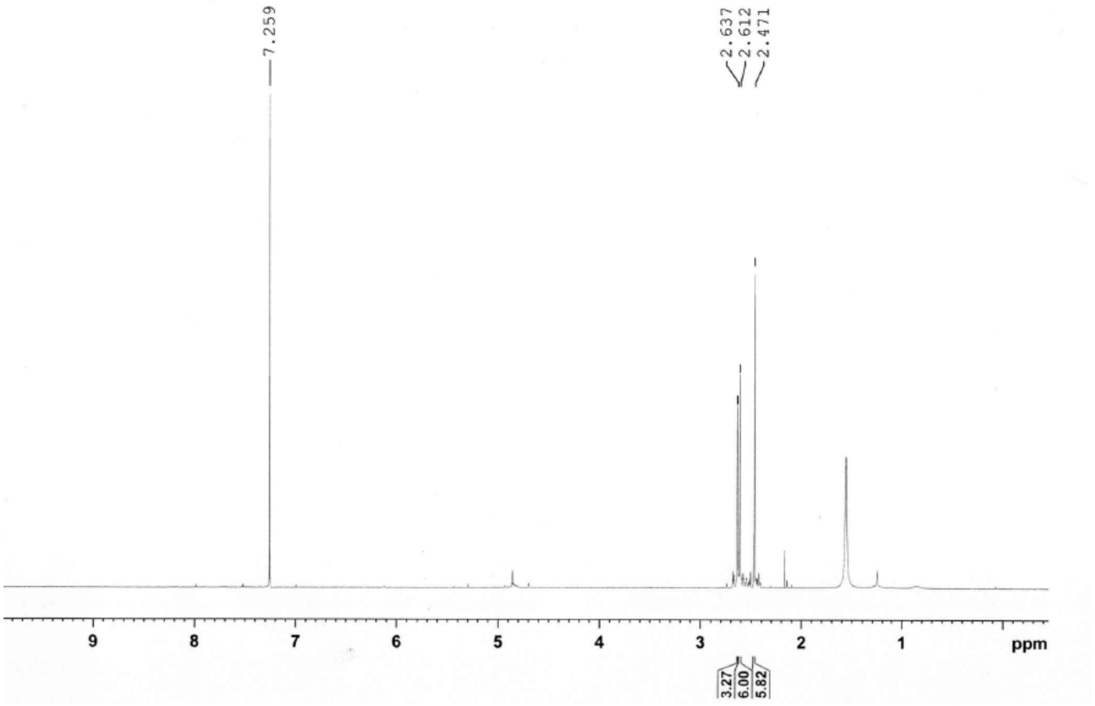

Figure S1: ^{1}H-NMR of 2,6-diiodo-BODIPY

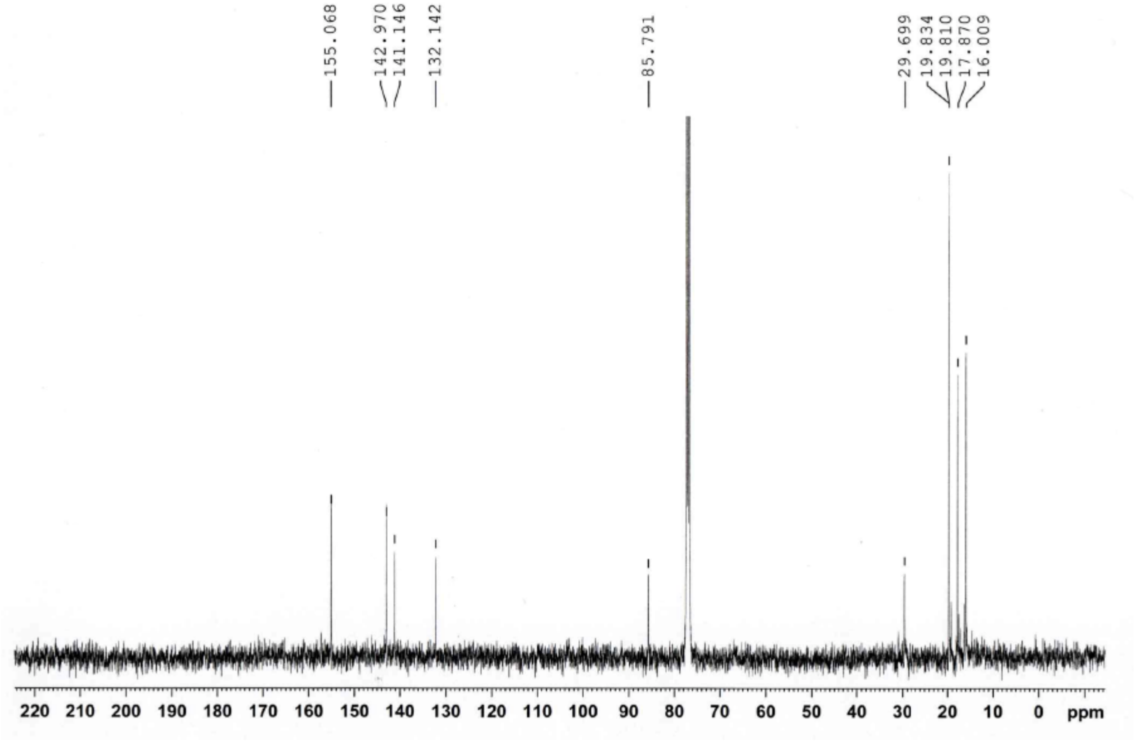

Figure S2: ^{13}C-NMR of 2,6-diiodo-BODIPY

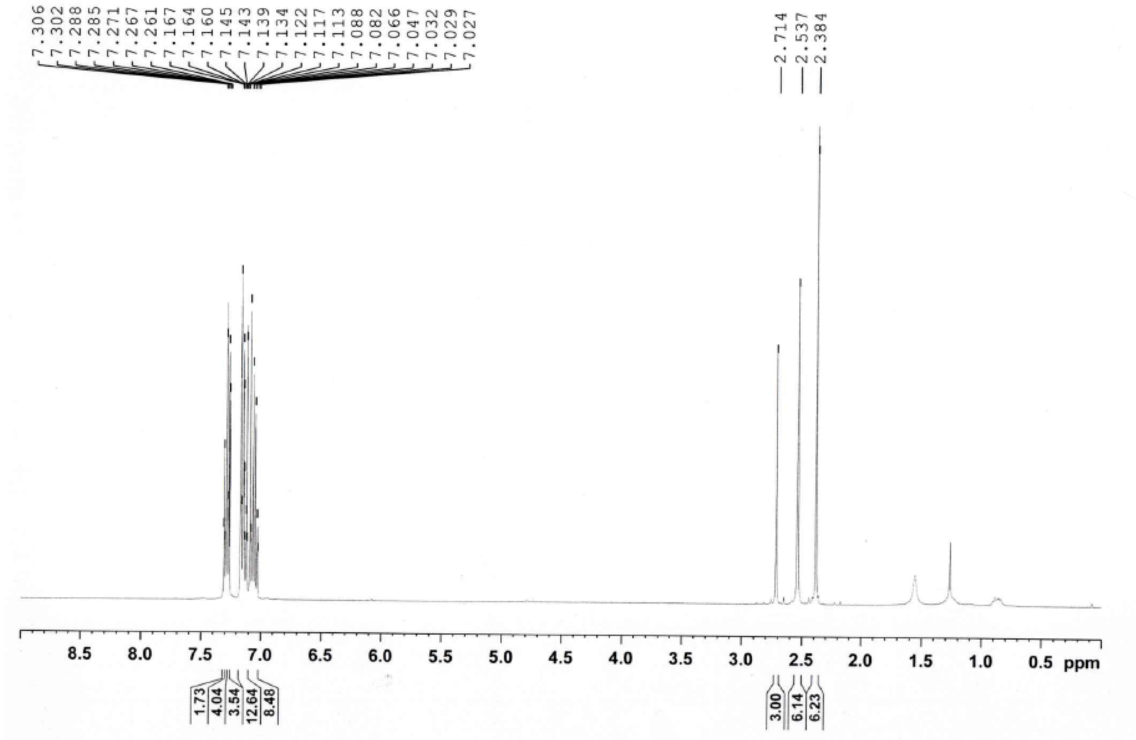

Figure S3: ^{1}H-NMR of **BODIPY-1** (**TPA-BODIPY-TPA**)

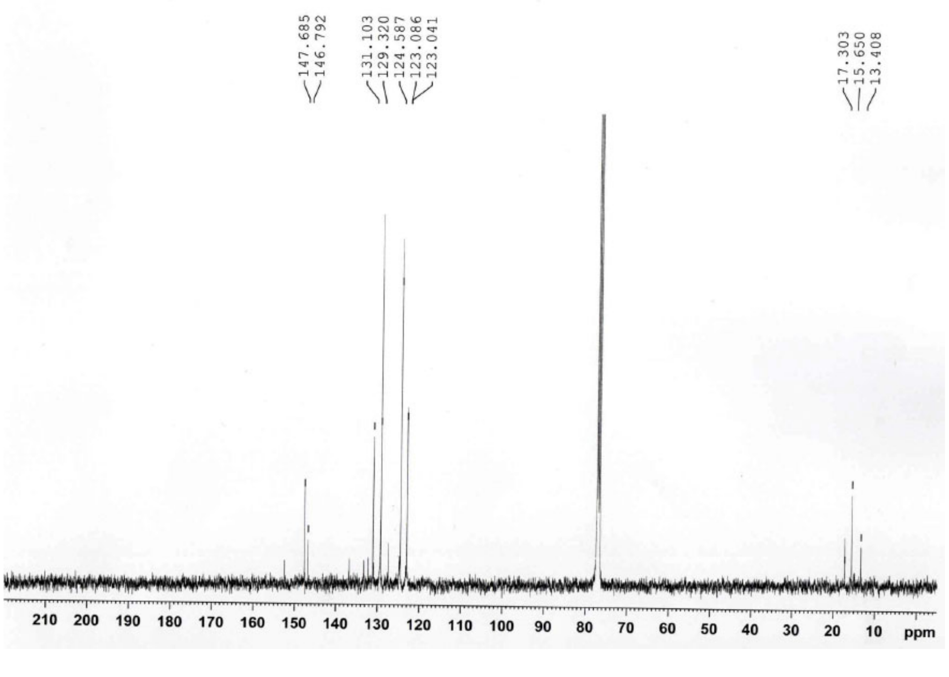

Figure S4: ^{13}C-NMR of **BODIPY-1** (**TPA-BODIPY-TPA**)

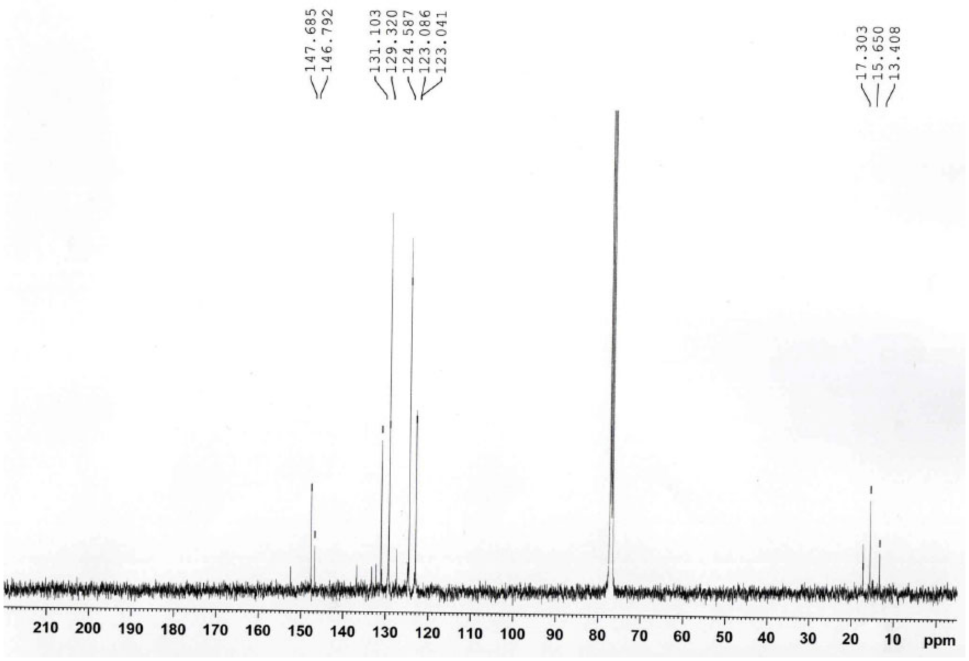

Figure S5: ^{1}H-NMR of **BODIPY-2** (**CBZ-BODIPY-CBZ**)

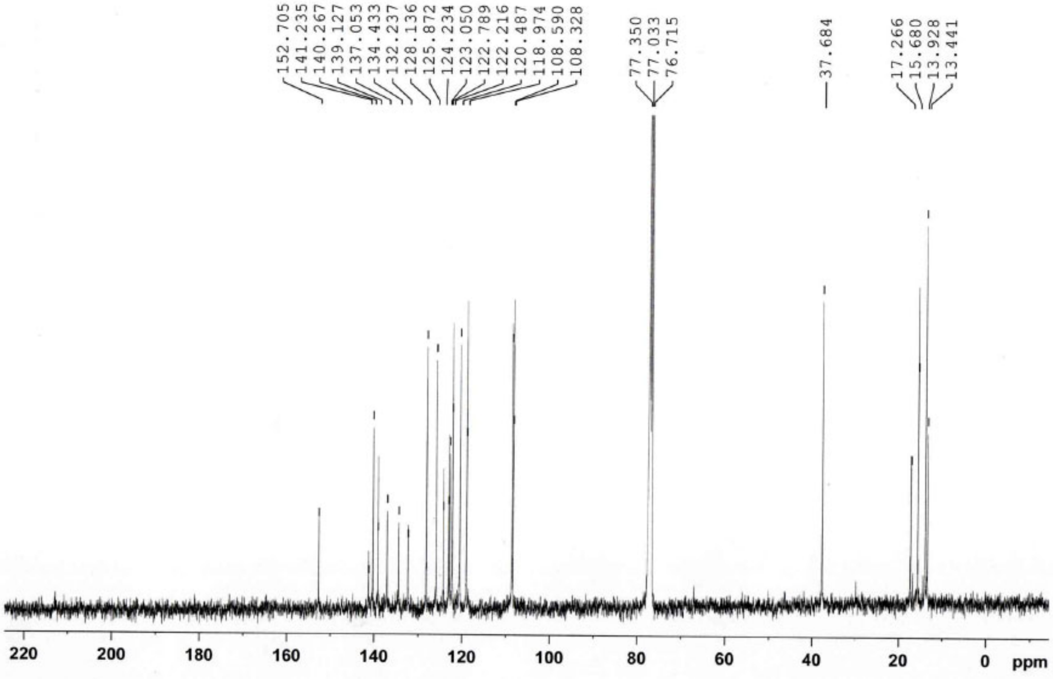

Figure S6: ^{13}C-NMR of **BODIPY-2** (**CBZ-BODIPY-CBZ**)

Thermal Gravimetric Analysis

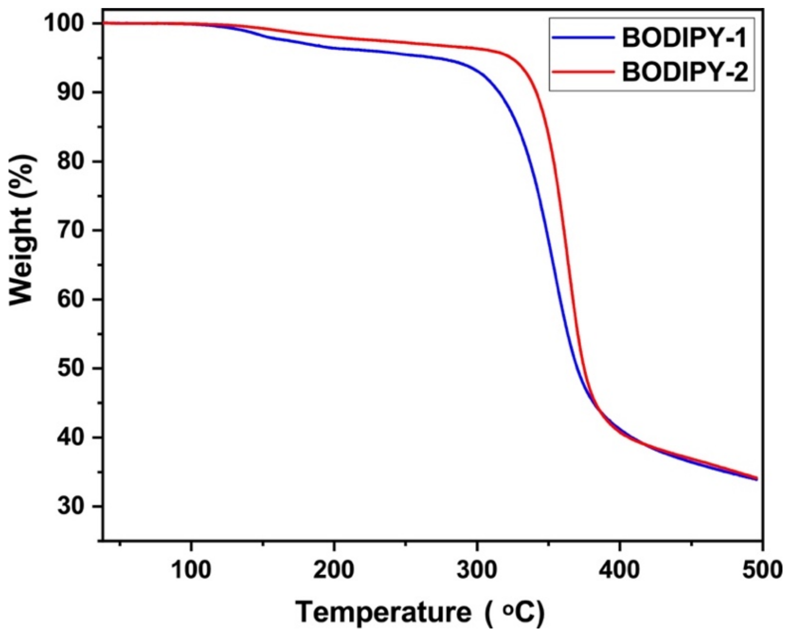

Figure S7: TGA of **BODIPY-1** and **BODIPY-2**

Photovoltaics Parameter

Table S1: Performance parameters of ternary solar cells with BODIPY:P3HT:PCBM (P3HT concentration was higher than BODIPY)

Devices	Donor ratio (by wt.)	J_{sc} (mA/cm^2)	V_{oc} (V)	FF	PCE (%)
1	**BODIPY-1**:P3HT (0.2:0.8)	4.15 ± 0.08	0.64 ± 0.03	49.90 ± 0.31	0.79 ± 0.04
2	**BODIPY-1**:P3HT (0.3:0.7)	4.07 ± 0.06	0.732 ± 0.01	34.69 ± 0.33	1.02 ± 0.02
3	**BODIPY-1**:P3HT (0.4:0.6)	3.96 ± 0.12	0.78 ± 0.03	34.69 ± 0.51	1.07 ± 0.02
4	**BODIPY-1**:P3HT (0.5:0.5)	4.03 ± 0.43	0.85 ± 0.03	41.82 ± 0.48	1.43 ± 0.12
5	**BODIPY-2**:P3HT (0.2:0.8)	4.02 ± 0.04	0.62 ± 0.01	29.67 ± 0.42	0.74 ± 0.01
6	**BODIPY-2**:P3HT (0.3:0.7)	4.13 ± 0.16	0.63 ± 0.01	29.53 ± 0.55	0.77 ± 0.06
7	**BODIPY-2**:P3HT (0.4:0.6)	4.70 ± 0.29	0.67 ± 0.02	30.84 ± 0.26	0.96 ± 0.04
8	**BODIPY-2**:P3HT (0.5:0.5)	3.88 ± 0.23	0.821 ± 0.02	41.16 ± 0.39	1.30 ± 0.13

The average values were obtained from 8 devices.

Table S2 Parameters fitted from the Nyquist plot of binary solar cells and ternary solar cells

Devices	R_s (Ω)	R_{rec} (Ω)	CPE-T (F/cm^2)	CPE-P
**BODIPY-1**	7.331	5462.80	3.38×10^{-5}	0.856
**BODIPY-2**	7.563	5718.20	3.20×10^{-5}	0.846
P3HT	7.859	6833.80	1.98×10^{-5}	0.704
**BODIPY-1:**P3HT (0.6:0.4)	7.464	9872.70	3.84×10^{-5}	0.903
**BODIPY-2:**P3HT (0.7:0.3)	7.459	7760.70	3.52×10^{-5}	0.903

Table S3: Contact Angle measurement of P3HT, **BODIPY-1**, **BODIPY-2** and PCBM

Compounds	Contact Angles
P3HT	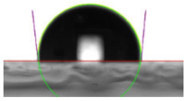 95.3
TPA-BODIPY-TPA (**BODIPY-1**)	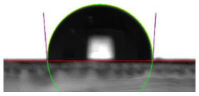 93.6
CBZ-BODIPY-CBZ (**BODIPY-2**)	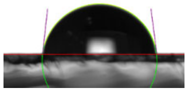 82.4
$PC_{61}BM$	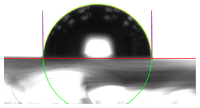 89.9
